# The Waite Testimonial Fund

**Published:** 1887-05

**Authors:** 


THE WAITE TESTIMONIAL FUND.

Some time since it was announced that Dr. W. H. Waite, of Liverpool, England, had been obliged to retire from practice on account of his loss of sight. Dr. Waite is well known in America, and the deep sympathy that was felt for him in his affliction was not confined to his English brethren. He is a graduate of an American Dental College, and has, at different times, made visits to this country, where, on account of his high character as a man and his excellent reputation as a dentist, he has always been warmly welcomed.

He has for many years been identified with dental progress, and has long and faithfully served the Midland Branch of the British Dental Association, as its Secretary. To mark their appreciation of him as a man, and to express their approbation of his long and faithful service in society matters, as well as to afford him some assistance in his hour of affliction, his English admirers determined to present him with an address and a purse of gold. The success of the movement was assured from the first by the willingness to serve upon the committee of such men as Sir Edwin Saunders, Sir John Tomes, J. S. Turner, Chas. S. Tomes, Morton Smale, Felix Weiss, Thos. Gaddes and others of the most illustrious of English Dentists.

The editor of this journal saw a notice of the appointment of the committee, and immediately wrote to the editor of *The Journal of the British Dental Association*, enquiring if American contributions would be compatible with the objects of the promoters of the fund, and expressing the firm conviction that, if an opportunity could be afforded, many Americans would feel a pleasure in subscribing. He enclosed in the letter a communication to the Treasurer of the fund. An answer was very promptly returned from Mr. Arthur S. Underwood, another from Mr. Renshaw, the Secretary of the fund, and a third from Dr. Waite himself. A meeting of the committee had been hastily convened, and the opinion was unanimous that such an expression of sympathy would do more to draw together the dentists of England and America than anything which could occur, and the only regret was that America had got the start of England in an expression of international courtesy and generosity. An editorial of Mr. Underwood, in *The Journal of the British Dental Association,* said: "We cannot imagine anything more gratifying to the object of the testimonial than the proposed graceful expression of genuine and heartfelt sympathy from America."

But the letters also conveyed the information that the presentation would be on April 29th, and the fund must be closed before that time. As the letters were not received until April 5th, there were but ten days for work before the contributions of American dentists must be forwarded. There was no time for consultation or combined action. If anything was to be done it must be without delay. We therefore determined to assume the responsibility, and make the appeal directly. A circular was prepared and sent out to but a few of the prominent dentists of America, with the request to send whatever they desired to subscribe at once.

We were quite aware that it was a great assumption on our part to presume to speak for our American brethren, but the circumstances of the case were an excuse. Had time permitted and a systematic effort been made, there is no doubt that the sum might have been more than doubled. As it is, there will probably be many who will feel a disappointment because they were not asked to contribute. We can only assert that we did not intend to exercise any partiality in sending out the circulars. We simply mailed them to those whose names came to memory, and nearly every one made a prompt, liberal and cheerful response. We did not ask for any sum larger than five dollars, and sent out but little more than fifty circulars.

When the responses to the circular began to arrive in such numbers that a measurable success was assured, we sent an ineffectual letter to one who has a better right to speak for American dentists than have we, inviting him to take charge of the fund and transmit it to the Hon. Treas. of the English fund, in the name of the dentists of America. The amount received is Four Hundred Dollars, which, as Prof. Taft declined the pleasant duty, we have remitted to England, with a letter explaining the circumstances under which it was raised. Several subscriptions which have since been received are now held, awaiting the orders of the subscribers.

In closing the matter up, we desire to thank those who so promptly responded, and to assure them that we firmly believe they will never see cause to regret their liberality.

The following is a list of the American subscribers to the fund, arranged in the order in which their contributions were received.

LIST OF AMERICAN SUBSCRIBERS TO THE WAITE TESTIMONIAL FUND.

| | | | |
|---|---|---|---|
| J. N. Farrar, New York City.. | $25 00 | W. F. Litch, Philadelphia..... | $ 5 00 |
| Frank Abbott, New York City. | 10 00 | C. N. Pierce, Philadelphia..... | 5 00 |
| Wm. Jarvie, Brooklyn, N. Y.. | 5 00 | James McManus, Hartford, Ct. | 20 00 |
| A. W. Harlan, Chicago, Ill.... | 10 00 | W. W. Allport, Chicago, Ill... | 25 00 |
| W. G. A. Bonwill, Philadelphia | 5 00 | H. A. Smith, Cincinnati, O.... | 5 00 |
| J.W.White,Phila.(S.S.W.D.M.Co.) | 25 00 | E. T. Darby, Philadelphia..... | 5 00 |
| F. J. S. Gorgas, Baltimore, Md. | 5 00 | James Truman, Philadelphia.. | 5 00 |
| George L. Field, Detroit, Mich. | 5 00 | T. T. Moore, Columbia, S. C... | 5 00 |
| Louis Jack, Philadelphia...... | 10 00 | C. F. W. Bodecker, N. Y. City. | 10 00 |
| J. L. Williams, Boston........ | 10 00 | Charles Barnes, Syracuse, N.Y. | 5 00 |
| S. B. Palmer, Syracuse, N. Y.. | 5 00 | A. O. Rawls, Lexington, Ky... | 5 00 |
| S. H. Guilford, Philadelphia... | 5 00 | F. H Rehwinkel Chilicothe, O. | 5 00 |
| C. D. Cook, Brooklyn, N. Y... | 10 00 | R.R.Andrews,Cambridge,Mass. | 5 00 |
| C.T Stockwell,Springfield Mass. | 2 50 | W. H. Morgan, Nashville, Tenn. | 5 00 |
| C. A Brackett, Newport, R. I. | 5 00 | A. H. Thompson, Topeka, Kan. | 2 50 |
| R. B. Winder, Baltimore, Md.. | 5 00 | J.H.Spaulding,Minneapolis,Minn. | 5 00 |
| W. N. Morrison, St. Louis, Mo. | 1 00 | T. B Wheeler, Chicago, Ill... | 5 00 |
| L. D. Shepard, Boston, Mass... | 10 00 | K. B. Davis, Springfield, Ill... | 5 00 |
| C. E. Francis, New York City. | 5 00 | J. Smith Dodge, Jr., N. Y. City | 5 00 |
| J.D.Patterson, Kansas City, Mo. | 2 00 | E. A. Bogue, New York City.. | 20 00 |
| W. B. Ames, Chicago, Ill..... | 5 00 | A. M. Dudley, Salem, Mass.... | 5 00 |
| Benj. Lord, New York City.... | 20 00 | J. Taft, Cincinnati, Ohio ..... | 25 00 |
| W.H.Dorrance,AnnArbor,Mich. | 2 00 | J. Morgan Howe,New York City | 10 00 |
| J. Bond Littig, New York City. | 10 00 | William Carr, New York City. | 10 00 |

The INDEPENDENT PRACTITIONER—Account of Printing, Postage, Collections, Stationery, Telegraphing, etc.........................

Total.............................................$400 00

